# The MoLfa1 Protein Regulates Fungal Development and Septin Ring Formation in *Magnaporthe oryzae*

**DOI:** 10.3390/ijms25063434

**Published:** 2024-03-19

**Authors:** Jia-Qi Wu, Xue-Ming Zhu, Jian-Dong Bao, Jiao-Yu Wang, Xiao-Ping Yu, Fu-Cheng Lin, Lin Li

**Affiliations:** 1 Zhejiang Provincial Key Laboratory of Biometrology and Inspection & Quarantine, College of Life Sciences, China Jiliang University, Hangzhou 310018, China; 15605398347@163.com (J.-Q.W.); yxp@cjlu.edu.cn (X.-P.Y.); fuchenglin@zju.edu.cn (F.-C.L.); 2 State Key Laboratory for Managing Biotic and Chemical Treats to the Quality and Safety of Agro-Products, Institute of Plant Protection and Microbiology, Zhejiang Academy of Agricultural Sciences, Hangzhou 310021, China; 11816090@zju.edu.cn (X.-M.Z.); baojd@zaas.ac.cn (J.-D.B.); wangjiaoyu78@sina.com (J.-Y.W.)

**Keywords:** *Magnaporthe oryzae*, septin ring, long-chain fatty acids (LCFAs), phosphatidylinositol phosphates (PIPs), pathogenicity

## Abstract

Septins play a key regulatory role in cell division, cytokinesis, and cell polar growth of the rice blast fungus (*Magnaporthe oryzae*). We found that the organization of the septin ring, which is essential for appressorium-mediated infection in *M. oryzae*, requires long-chain fatty acids (LCFAs), which act as mediators of septin organization at membrane interfaces. However, it is unclear how septin ring formation and LCFAs regulate the pathogenicity of the rice blast fungus. In this study, a novel protein was named MoLfa1 because of its role in LCFAs utilization. MoLfa1 affects the utilization of LCFAs, lipid metabolism, and the formation of the septin ring by binding with phosphatidylinositol phosphates (PIPs), thereby participating in the construction of penetration pegs of *M. oryzae*. In addition, MoLfa1 is localized in the endoplasmic reticulum (ER) and interacts with the ER-related protein MoMip11 to affect the phosphorylation level of Mps1. (Mps1 is the core protein in the MPS1-MAPK pathway.) In conclusion, MoLfa1 affects conidia morphology, appressorium formation, lipid metabolism, LCFAs utilization, septin ring formation, and the Mps1-MAPK pathway of *M. oryzae*, influencing pathogenicity.

## 1. Introduction

Rice blast fungus (*Magnaporthe oryzae*) is a plant pathogenic fungus that severely affects grain growth and development and reduces production. In recent years, rice blast has spread to more than 85 countries, causing an annual decline in rice yield of about 30%, with severe epidemics resulting in losses of up to 80% of the rice crop yield [1]. Septins mediate *M*. *oryzae*’s invasion of plant cells. Deletion of the core septin genes *MoSEP3*, *MoSEP4*, *MoSEP5*, and *MoSEP6* resulted in attenuated pathogenicity in *M. oryzae* [1]. Septins are a class of conserved guanosine triphosphate GTP-binding proteins [2]. The septin family of proteins was identified in a screen for cell division regulatory genes in *Saccharomyces cerevisiae* [3]. Septins can assemble into symmetrical linear heterooligomeric complexes. These complexes can polymerize into higher-order structures (such as filamentous fibers or hourglass rings) at the division plane or polar location with the plasma membrane [4], where they mainly act as scaffolding proteins and diffusion barriers in cell division, cytokinesis, sperm, and ciliogenesis, and play a key regulatory role in cell polar growth and other life activities [5,6,7,8]. Septin forms filamentous structures similar to microfilaments, microtubules, and microfibers in mammalian cells and is therefore considered the fourth type of cytoskeleton [6]. Septin proteins interact with the middle GTP binding domain via their C-terminal and N-terminal, first forming a mirror-symmetric oligomeric complex and then polymerizing to form filamentous fibers, hourglasses, rings, and other advanced structures [9]. Most N-terminal septin proteins contain a polybasic region (PBR) that can interact with negatively charged PIPs and bind to the cell membrane [5]. Cdc3, Cdc10, Cd11, and Cdc12 are the core septin genes in *S. cerevisiae*. In yeast, four septin subunits form Cdc11-Cdc12-Cdc3-Cdc10-Cdc10-Cdc3-Cdc12-Cdc11 heterooctamer rods that are polymerized into filaments and closely contact the inner surface of the plasma membrane to form a ring structure [5]. In *M. oryzae*, Sep3, Sep4, Sep5, and Sep6 are homologous to Cdc3, Cdc10, Cdc11, and Cdc12 in yeast. PIPs mediate the enrichment of septins at specific membrane regions to specialize cell shape. In *M. oryzae*, septins generate cell polar growth, which enables the *M. oryzae* appressoria to produce penetration pegs for host infection [1,10].

Lipids are a large class of chemically and structurally heterogeneous compounds, mainly including fatty acids and their naturally occurring derivatives (such as esters or amines), as well as compounds related to their biosynthesis and function. As a significant lipids component, glycerophospholipids (GPs) play multiple roles in cellular processes, including membrane structure formation, cell signaling, membrane trafficking, and membrane protein anchoring [11,12,13]. GPs mainly consist of phosphatidylinositol (PI), phosphatidylserine (PS), phosphatidylethanolamine (PE), cardiolipin (CL), and phosphatidylcholine (PC) in yeast, all derived from the precursor lipid phosphatidic acid (PA) [14]. PIPs are phosphorylated forms of PI generated by various PI and PIPs kinases. In eukaryotic cells, PIPs play a crucial role in almost every cellular process [15]. PIPs are precursors of second messengers that regulate ion channels and transport proteins [16,17]. PIPs are the major regulators of vesicle trafficking, secretion, and endocytosis, and they also play critical roles in lipid transport processes [15,18,19]. Recent studies have shown that PIPs associate with septins to aggregate at cell membranes, regulating various membrane-associated cellular processes required for cell shape control [10,20,21]. LCFAs are fatty acids with more than 12 carbon atoms and are an important component of PIPs. LCFAs have essential biological roles as nutrients and substrates for signaling molecules [22]. LCFAs also play crucial roles in physiological and pathological processes. They are activators and inhibitors of energy sources, enzymes, and membrane proteins and are involved in energy, glucose, and lipid metabolism [23,24,25]. Furthermore, LCFAs are not only an essential component of PIPs but also possess unique biophysical properties such as increasing lipid hydrophobicity, participating in the formation of lipid blamers, and mediating the transition of lipids from the fluid to the gel phase [26,27]. These unique properties enable LCFAs to promote high bending of membrane domains and maintain their stability [26]. Recent studies have reported that LCFAs can regulate the pathogenicity of *M. oryzae* by mediating septin ring formation [10].

In *M. oryzae*, appressorium formation, penetration pegs emergence, and other infection-related processes are regulated by many conserved signaling pathways, such as those mediated by three mitogen-activated protein kinases (MAPK) [28,29,30]. The Pmk1, Mps1, and Osm1 MAPK signaling pathways are activated by a core kinase cascade that adapts to different transduction stress conditions [31,32]. In the Mps1-MAPK pathway, which is derived from the Bck1-Mkk1/Mkk2-Slt2 signaling pathway in yeast, Mck1 and Mkk2 are MEKK [33], and MEK acts upstream of Mps1, and both Mck1 and Mkk2 can interact with the bridging protein Mst50 of the Mst11-Mst7-Pmk1 cascade [34]. In addition, Mps1 directly interacts with two important downstream transcription factors, MoMig1 and MoSwi6 [35]. Loss of both *MoMIG1* and *MoSWI6* causes defects in fungal development [33]. A recent study also found that the RACK1 homologous protein MoMip11 in *M. oryzae* is mainly located in the ER and regulates a variety of cell functions and developmental pathways, including vegetative growth, conidium formation, appressorium formation, Mps1 phosphorylation level, and pathogenicity. The Δ*Momip11* mutant has severe defects in cell wall integrity and plant infection [36].

In this study, we found a novel protein that has homologous proteins only in pathogenic fungi such as *Fusarium graminearum*, *Fusarium oxysporum*, *Blumeria graminis* f. sp*. tritici*, *Colletotrichum* spp., and some endophytic fungi, but no homologous proteins in yeast, humans, mice, drosophila, or nematodes. Because of its role in LCFAs utilization, we named it MoLfa1. We discovered that MoLfa1 is involved in mycelial growth, appressorium formation, lipid droplet utilization, and the pathogenicity of *M. oryzae*. In addition, MoLfa1 is shown to be mainly concentrated in the ER and to interact with the ER-related protein MoMip11 to regulate the Mps1 phosphorylation level, thereby affecting pathogenicity. More importantly, we discovered that MoLfa1 is involved in lipid metabolism and LCFAs absorption and can bind to PIPs to interfere with the normal septin ring formation, thus affecting the appearance of penetration pegs and pathogenicity.

## 2. Results

### 2.1. Identification of MoLfa1 in M. oryzae

In the data of *M. oryzae*, taken from the EnsemblFungi database (http://fungi.ensembl.org/Magnaporthe_oryzae/Info/Index?db=core, accessed on 10 July 2021), we found a gene with ID number MGG_05194, which we named MoLfa1. MoLfa1 has no homologous protein in yeast, humans, mice, drosophila, or nematodes. Still, it has a high degree of homology with other pathogenic fungi and some endophytic fungi, including CTRU02_14685 of *Colletotrichum truncatum* (53.7% identity), FOYG_11232 of *F. oxysporum* NRRL 32931 (53.4% identity), FGSG_05529 of *F. graminearum* PH-1 (53.1% identity), BCIN_08g03690 of *Botrytis cinerea* B05.10 (45.1% identity), BGT96224_3476 of *B. graminis* f. sp. *tritici* 96224 (38.3% identity), N656DRAFT_704297 of *Canariomyces arenarius* (57.2% identity), GGTG_01025 of *Gaeumannomyces tritici* R3-111a-1 (60.3% identity) and F4806DRAFT_12431 of *Annulohypoxylon nitens* (58.7% identity) (Figure S1A). Homology analysis revealed that MoLfa1 and the above homologous proteins are genetically close, indicating that they are highly conserved in evolution (Figure S1B). Protein structure prediction showed that MoLfa1 contains a disordered region (95–122aa), and the model confidence in this region is very low (Figure S1C).

### 2.2. MoLfa1 Is Involved in the Growth, Conidia Development, and Appressorium Formation of M. oryzae

**Figure 1:**
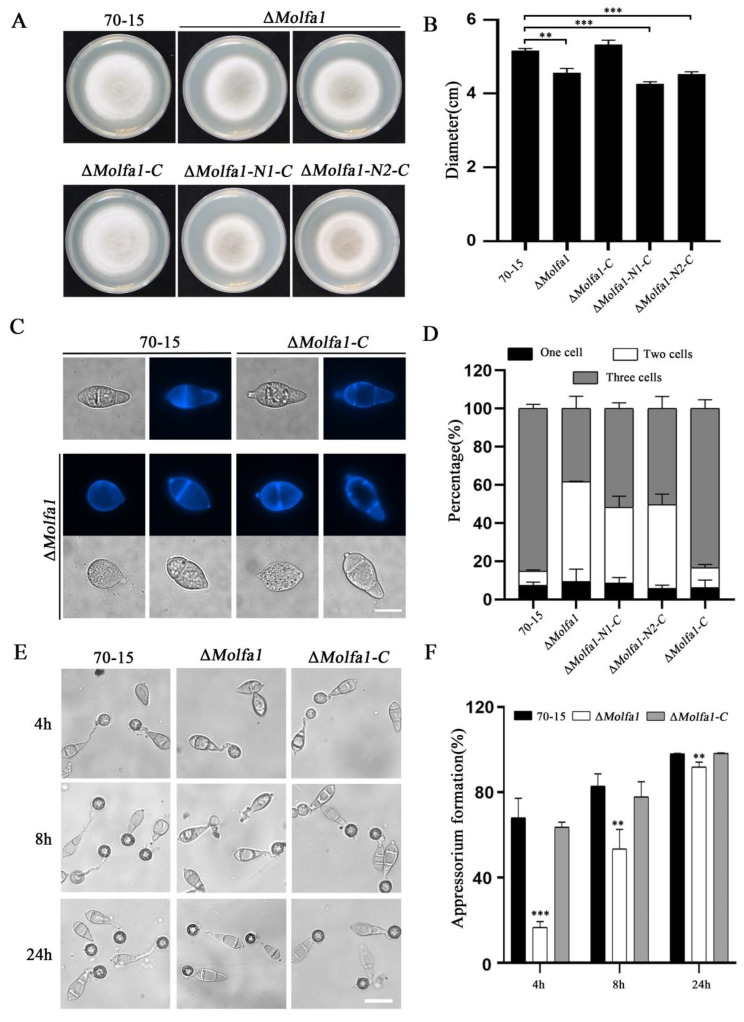
MoLfa1 is involved in the growth, conidial development, and appressorium formation of *M. oryzae.* (**A**) The morphology of wild-type 70-15, the Δ*Molfa1* mutant, complement strain Δ*Molfa1-C*, Δ*Molfa1-N1-C*, and Δ*Molfa1-N2-C* after 8 days of culture at 25 °C in CM. (**B**) Colony diameters of wild-type 70-15, the Δ*Molfa1* mutant, Δ*Molfa1-C* complement strains, Δ*Molfa1-N1-C*, and Δ*Molfa1-N2-C* complement strains in CM were statistically analyzed. An asterisk indicates a significant difference (*t*-test, ** *p* < 0.01, *** *p* < 0.001). (**C**) Morphology of the conidia of wild-type 70-15, the Δ*Molfa1* mutant, complement strain Δ*Molfa1-C* andΔ*Molfa1-N1-C* and Δ*Molfa1-N2-C* strains. Scale, 10 μm. (**D**) conidia anomaly rates in wild-type 70-15, the Δ*Molfa1* mutant, complement Δ*Molfa1-C* strain, Δ*Molfa1-N1-C*, and Δ*Molfa1-N2-C*. (**E**) Appressorium formation of 70-15, the Δ*Molfa1* mutant, complement strain Δ*Molfa1-C* was observed on the surface of the hydrophobic membrane. Scale, 20 μm. (**F**) Statistical analysis of appressorium formation rates (%) of wild-type 70-15, the Δ*Molfa1* mutant, and Δ*Molfa1-C* at 4 h, 8 h and 24 h of induction (*t*-test, ** *p* < 0.01, *** *p* < 0.001).

To explore the function of Molfa1 in *M. oryzae*, we transferred the constructed hygromycin-resistance gene (*HPH*) knockout vector into the wild-type strain 70-15. We successfully obtained a Δ*Molfa1* deletion mutant (Figure S2). To investigate the biological function of MoLfa1 in *M. oryzae*, we used the full-length *MoLFA1* gene in the PKD5-GFP vector containing a sulfonylurea-resistance gene (*SUR*) and transformed it into the Δ*Molfa1* mutant by an *Agrobacteria*-mediated transformation method (ATMT) [37] to obtain the complementation strain Δ*Molfa1-C*. To study the influence of the disordered region on MoLfa1 function, the full-length *MoLFA1* gene was split into two segments. Since the MoLfa1 protein contains a disordered region (283–366 bp contains the disordered region), we split its sequence into two segments, N1 (1–282 bp) and N2 (283–468 bp). We then fused the segments into the Δ*Molfa1* mutant to obtain the Δ*Molfa1-N1* and Δ*Molfa1-N2* strains, respectively. When the strain was inoculated at 25 °C above complete medium (CM) for 9 days, the colony diameter of the Δ*Molfa1* mutant was significantly smaller compared to that of wild-type 70-15 and Δ*Molfa1-C*. The colony diameters of Δ*Molfa1-N1-C* and Δ*Molfa1-N2-C* also did not reach normal levels but were similar to those of the Δ*Molfa1* mutant (Figure 1A,B). We also observed the conidial morphology of the strains cultured on CM for 8 days. The conidia of wild-type 70-15 and Δ*Molfa1-C* mainly were three cells, whereas about 50% of the conidia produced by the Δ*Molfa1* mutant were two cells. The conidia of Δ*Molfa1-N1-C* and Δ*Molfa1-N2-C* were also predominantly two-cell conidia, similar in morphology to those of the Δ*Molfa1* mutant (Figure 1C,D). Although the abnormal rate of the Δ*Molfa1* mutant conidia was significantly increased, conidia germination was unaffected. The rate and speed of appressorium formation on the hydrophobic surface of the Δ*Molfa1* mutant conidia was significantly reduced compared to wild-type 70-15 and Δ*Molfa1-C*. After 4 h of induction, only 16.65% of the Δ*Molfa1* mutant conidia formed appressoria, whereas the appressorium formation rate in wild-type 70-15 was approximately 68.08%. After 8 h of induction, the appressorium formation rate of the Δ*Molfa1* mutant (53.43%) was significantly lower than that of wild-type 70-15 (82.96%). Even after 24 h of induction, the appressorium formation rate of the Δ*Molfa1* mutant was significantly different from that of wild-type 70-15 (Figure 1E,F). To sum up, MoLfa1 is involved in the growth, conidial development, and appressorium formation of *M. oryzae*.

### 2.3. Subcellular Localization of MoLfa1

**Figure 2:**
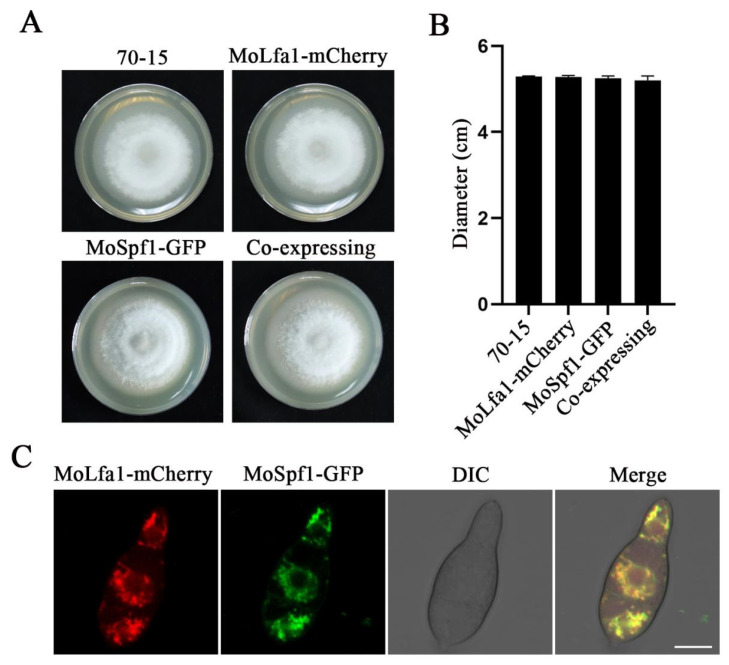
Subcellular localization of MoLfa1. (**A**) Colony morphology of wild-type 70-15, MoLfa1-mCherry expressing strain, MoSpf1-GFP expressing strain, and MoLfa1-mCherry and MoSpf1-GFP co-expressing strain. (**B**) Colony diameters of wild-type 70-15, MoLfa1-mCherry expressing strain, MoSpf1-GFP expressing strain, and MoLfa1-mCherry and MoSpf1-GFP co-expressing strain. (**C**) Fluorescence images of conidia co-expressing MoLfa1-mCherry and MoSpf1-GFP. Scale, 10 μm.

To understand the function of MoLfa1 in *M. oryzae*, subcellular localization of MoLfa1 was performed. We constructed a *MoLFA1*-mCherry expression vector and transferred it into the Δ*Molfa1* mutant by ATMT transformation. As shown in Figure 2A,B, the strain expressing MoLfa1-mCherry was similar in morphology and diameter to the wild-type 70-15, indicating that MoLfa1-mCherry could be expressed normally. Under fluorescence microscopy, MoLfa1-mCherry showed a filamentous distribution in conidia. Therefore, we speculated that MoLfa1-mCherry was mainly located in the ER. To further determine the subcellular localization of MoLfa1, we transferred the ER marker gene *MoSPF1*-GFP into strains with MoLfa1-mCherry fluorescent vectors. Similarly, MoLfa1-mCherry and MoSpf1-GFP were expressed normally in the strains (Figure 2A,B). The conidia of co-expressing MoLfa1-mCherry and MoSpf1-GFP strains were observed by fluorescence microscopy. MoLfa1-mCherry could colocalize with the MoSpf1-GFP signal in each conidial cell (Figure 2C). The results showed that MoLfa1 is mainly located in the ER of *M. oryzae*.

### 2.4. MoLfa1 Is Involved in Lipid Metabolism in M. oryzae

**Figure 3:**
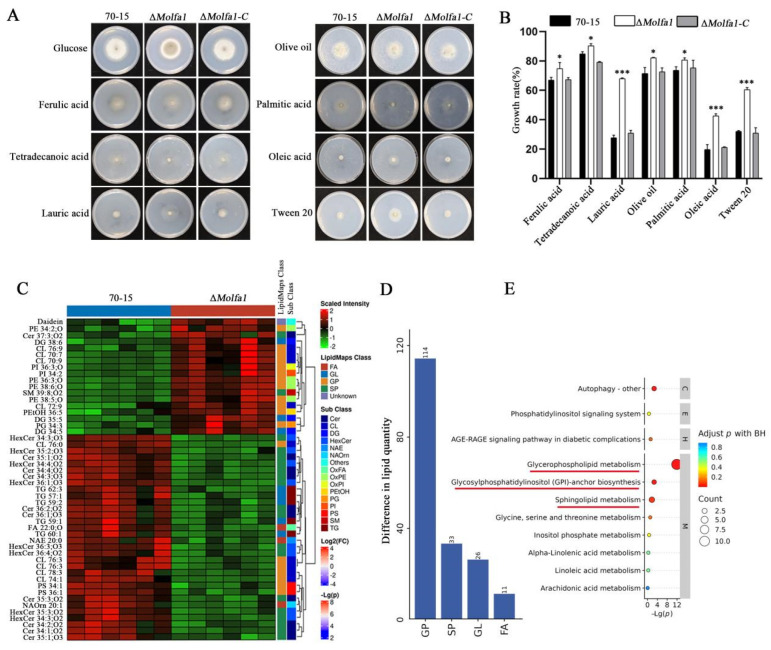
MoLfa1 is involved in lipid metabolism in *M. oryzae.* (**A**) Wild-type 70-15, the Δ*Molfa1* mutant, and Δ*molfa1-C* were grown on MM medium with carbon source only days in glucose, ferulic acid, tetradecanoic acid, lauric acid, palmitic acid, olive oil, oleic acid, and Tween 20 for 8 and photographed. (**B**) Wild-type 70-15, the Δ*molfa1* mutant, and Δ*molfa1-C* were grown on different LCFAs media for 8 days (*t*-test, * *p* < 0.05, *** *p* < 0.001). (**C**) Heat maps showing lipidomic analyses of wild-type 70-15 and the Δ*molfa1* mutant. Each rectangle represents a lipid colored by its normalized intensity scale from red (increased level) to green (decreased level). FA: fatty acyls; GL: glycerolipids; GP: glycerophospholipids; SP: sphingolipids. (**D**) Differential lipid maps of wild-type 70-15 and the Δ*molfa1* mutant. (**E**) Enrichment map of the KEGG pathway. The horizontal coordinate represents the negative logarithmic transformation of the *p* value, and the vertical coordinate represents the pathway class name. The red underline highlights the enrichment pathway. M: metabolism; E: environmental information processing; C: cellular processes; H: human diseases.

Since Molfa1 is located in the ER, which is the main site of LCFAs synthesis, we hypothesized that there is a relationship between Molfa1 and utilization of LCFAs. We replaced 1% of the carbon source glucose in MM with LCFAs, including ferulic acid (it has 20 carbon atoms, 20C), tetradecanoic acid (12C), lauric acid (12C), palmitic acid (16C), olive oil (18C), oleic acid (18C), and tween 20 (26C). Growth rates of wild-type 70-15, the Δ*Molfa1* mutant, and complement strains in different carbon sources were calculated using medium MM as a control. The utilization rate of LCFAs of the Δ*Molfa1* mutant was higher than that of wild-type 70-15. The ability of the Δ*Molfa1* mutant to utilize lauric acid, oleic acid, and tween 20 was significantly higher than that of the wild-type 70-15 (about twofold). The growth rates of the Δ*Molfa1* mutant were 74.79%, 90.31%, 80.59%, and 82.17% in ferulic acid, lauric acid, palmitic acid, and olive oil, respectively, which were significantly different from that of wild-type 70-15 (Figure 3A,B). The results showed that the absence of *MoLFA1* in *M. oryzae* affected the utilization rate of LCFAs. In particular, MoLfa1 significantly inhibited the utilization of lauric acid, oleic acid, and tween 20.

Since MoLfa1 is involved in the utilization of LCFAs, to further investigate the role of MoLfa1 in lipid utilization in *M. oryzae*, we performed non-targeted lipidomic analyses of wild-type 70-15 and the Δ*Molfa1* mutant [38]. In this experiment, 210 significant differences were screened. These significant differences were classified into four subclasses, namely fatty acyl (FA), GPs, sphingolipids (SP), and glycerides (GL). As shown in Figure 3D, the differential lipids of wild-type 70-15 and the Δ*Molfa1* mutant were approximately 61.69% GPs, 17.93% SP, 14.13% GL and 5.98% FA. We used the expression levels of lipids in the top 50 qualitatively significant differences to perform hierarchical clustering for each group of samples. The results showed an apparent hierarchical clustering between wild-type 70-15 and the Δ*Molfa1* mutant. Compared with the wild-type 70-15, 12 GPs, 3 GL, one unknown lipid, and 2 SP were significantly up-regulated, whereas 17 GL, 7 GP, 17 SP, 5 GL and 3 FA were significantly down-regulated in the Δ*Molfa1* mutant (Figure 3C). To investigate the pathway of differential expression of the Δ*Molfa1* mutant at the molecular level, we performed pathway enrichment analysis of differential lipids using the Lipid Pathway Enrichment Analysis (LIPEA) tool. A Kyoto Encyclopedia of Genes and Genomesenrichment (KEGG) analysis showed that the differential expression of lipid molecules in this experiment was mainly related to glycerophospholipid metabolism. It is also associated with sphingolipid metabolism and glycosylphosphatidyl (GPI)-anchor biosynthesis (Figure 3E). In conclusion, MoLfa1 is involved in lipid metabolism in *M. oryzae.*

### 2.5. MoLfa1 Can Associate with PIPs to Regulate the Formation of Septin Ring

**Figure 4:**
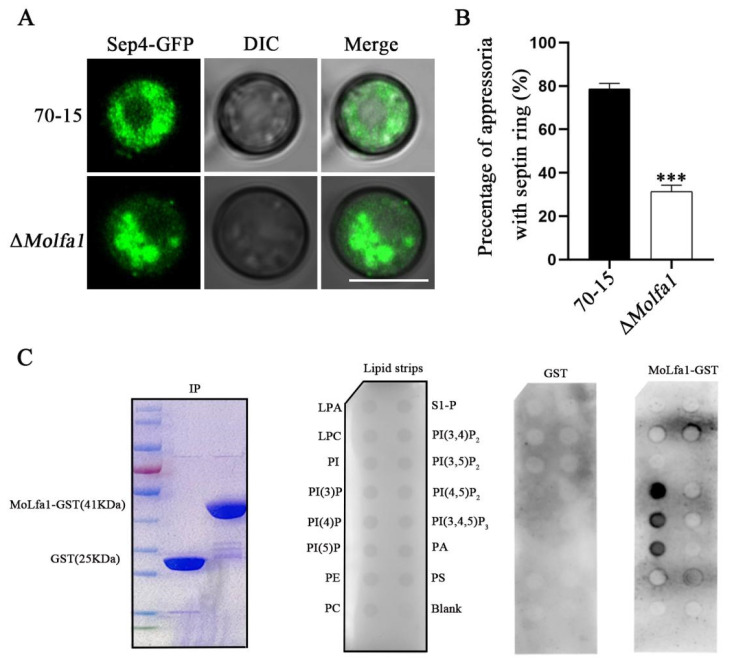
MoLfa1 can associate with PIPs to regulate septin ring formation. (**A**) Observation of the septin ring morphology in appressoria after 24 h induction of wild-type 70-15 and the Δ*Molfa1* mutant. Scale, 10 μm. (**B**) Statistical analysis of typical septin ring in wild-type 70-15 and the Δ*Molfa1* mutant 24 h after induction (*t*-test, *** *p* < 0.001). (**C**) In in vitro lipid binding experiments, MoLfa1 could bind PI3P, PI4P, and PI5P. The PIP strips of purified MoLfa1-GST and empty GST were incubated with 1 μg/mL skim milk powder solution. The anti-GST antibodies detected MoLfa1-GST and empty GST. LPA, lysophosphatidic acid; LPC, lysophosphocholine; PtdIns, phosphatidylinositol; PE, phosphatidylethanolamine; PC, phosphatidylcholine; S1-P, sphingosine-1-phosphate; PA, phosphatidic acid; PS, phosphatidylserine.

The septin ring is the critical structure for *M. oryzae* appressoria to infect the host successfully. In *M. oryzae*, the major components of the septin ring are MoSep3, MoSep4, MoSep5, and MoSep6. To verify the function of MoLfa1 in septin ring formation, we constructed the septin ring localization gene *MoSEP4*-GFP. We transferred it into wild-type 70-15 and the Δ*Molfa1* mutant by ATMT transformation and then observed and photographed them under the microscope. We found that the morphology of the Δ*Molfa1* mutant’s septin ring was different from that of the wild-type 70-15 (Figure 4A,B).

It has been reported that septin ring formation requires PIPs associated with septins to aggregate at cell membranes [10]. We found that MoLfa1 affects the utilization of LCFAs and the lipid metabolism of *M. oryzae*. PIPs consist of a phosphorylated inositol ring and two fatty acid chains linked by a glycerol master bond and are a type of GPs [15]. We speculated that MoLfa1 might have a potential relationship with PIPs, so we performed in vitro lipid binding experiments. It was found that MoLfa1 could bind phosphoinositol-3-phosphate (PI3P), phosphoinositol-4-phosphate (PI4P), and phosphoinositol-5-phosphate (PI5P) (Figure 4C). To sum up, MoLfa1 associates with PIPs, affects the formation of the septin ring, and then affects the penetration pegs’ formation of *M. oryzae*, thereby preventing its infection of rice.

### 2.6. MoLfa1 Is Essential for Pathogenicity

**Figure 5:**
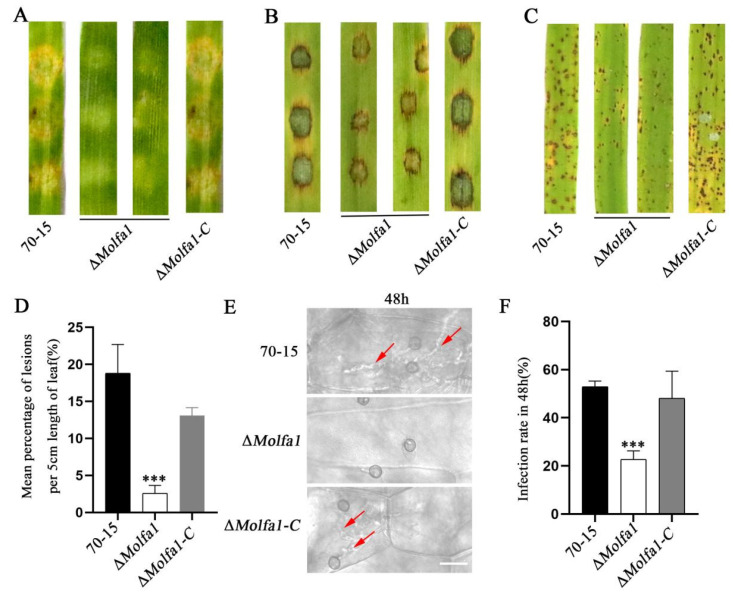
MoLfa1 is required for the pathogenicity of *M. oryzae*. (**A**) Pathogenicity assay on barley leaves. The wild-type 70-15, the Δ*Molfa1* mutant, and the complement strains Δ*Molfa1-C* were inoculated on cultured barley leaves. Typical leaves were photographed 4 days after inoculation. (**B**) Conidia were collected with sterile water and diluted to 1 × 10^5^/mL. 20 μL of conidial suspension was dropped on barley leaves and typical leaves were photographed 4 days after inoculation. (**C**) Conidia were collected with sterile water and then suspended in 0.25% gelatin to achieve a conidial concentration of 5 × 10^4^/mL. 2 mL conidial suspension was evenly sprayed on 14-day-old rice seedlings, and the formation of disease spots on rice leaves was assessed for 7 days after inoculation. (**D**) The number of lesions per 5 cm of rice leaves (*t*-test, *** *p* < 0.001). (**E**) Infection from isolated barley leaves 48 h after inoculation with conidia of wild-type 70-15, the Δ*Molfa1* mutant, and Δ*Molfa1-C*. The arrow points to an infectious hypha. Scale, 20 μm. (**F**) Rates of penetration pegs formation and invasive hyphae extended of wild-type 70-15, the Δ*Molfa1* mutant, and Δ*Molfa1-C* at 48 h (*t*-test, *** *p* < 0.001).

To further verify the role of MoLfa1 in the pathogenicity of *M. oryzae*, we performed pathogenicity assays on two susceptible hosts (barley and rice). Wild-type 70-15, the Δ*Molfa1* mutant, and Δ*Molfa1-C* strains growing on CM were attached to isolated barley leaves and cultured in moist conditions for 4 days. We discovered that Δ*Molfa1-C* and wild-type 70-15 caused severe leaf lesions, but leaves inoculated with the Δ*Molfa1* mutant showed significantly less pathogenicity (Figure 5A). The conidial suspensions of the three strains (5 × 10^4^/mL) were dropped on isolated barley leaves and cultured under moist conditions for 4 days. We found that leaves inoculated with the Δ*Molfa1* mutant showed smaller lesions than leaves inoculated with wild-types 70-15 and Δ*Molfa-1-C* (Figure 5B). Similarly, a suspension of conidia (5 × 10^4^/mL) was sprayed on susceptible rice seedlings (*Oryza sativa* cv. CO-39) for 7 days of culture. Small and isolated disease spots were found on rice leaves sprayed with Δ*Molfa1* mutant conidia, whereas typical fusiform disease spots were found on rice leaves sprayed with wild-type 70-15 and Δ*Molfa1-C* conidia (Figure 5C). Quantitative statistical analysis of the lesion areas (%) showed that the rice lesion area caused by the Δ*Molfa1* mutant was only 2.60%, which was seven times lower than that caused by wild-type 70-15 (18.78%) (Figure 5D). In addition, we observed the ability of the strains to infect rice by appressorium penetration experiments. The appressoria of 70-15 successfully penetrated plant cells, and the penetration pegs had expanded to form infectious hyphal branches. However, only about 20% of the appressoria successfully infected barley leaves inoculated with the Δ*Molfa1* mutant conidia (Figure 5E,F). In conclusion, MoLfa1 is required for the pathogenicity of *M. oryzae*.

### 2.7. Disruption of MoLFA1 Delays the Mobilization and Degradation of Glycogen and Lipid Droplets

**Figure 6:**
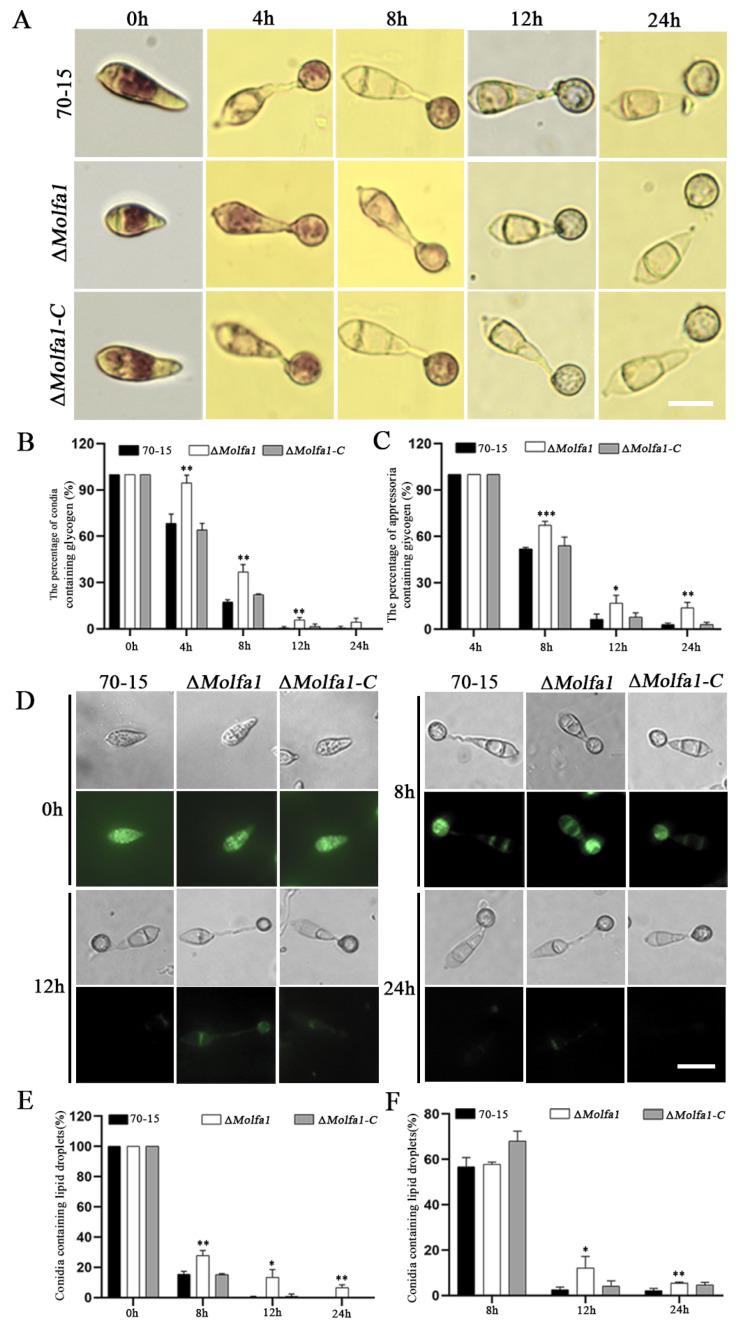
Disruption of *MoLFA1* delays the mobilization and degradation of glycogen and lipid droplets. (**A**) Glycogen distribution of wild-type 70-15, the Δ*Molfa1* mutant, and Δ*Molfa1-C* conidia and appressoria after 0 h, 4 h, 8 h, 12 h, and 24 h induction. The samples were stained with KI/I_2_. Glycogen appears as dark brown deposits under the microscope. Scale, 10 μm. (**B**) Percentage of glycogen-containing conidia of wild-type 70-15, the Δ*Molfa1* mutant, and Δ*Molfa1-C* (*t*-test, ** *p* < 0.01). (**C**) Percentage of glycogen-containing appressoria in wild-type 70-15, the Δ*Molfa1* mutant, and Δ*Molfa1-C* (*t*-test, * *p* < 0.1, ** *p* < 0.01, *** *p* < 0.001). (**D**) Lipid droplet distribution of conidia and appressoria of wild-type 70-15, the Δ*Molfa1* mutant, and Δ*Molfa1-C* after 0 h, 8 h, 12 h, and 24 h induction. The samples were stained with Bodipy. Lipid droplets appear as green fluorescence under a fluorescence microscope. Scale, 20 μm. (**E**) Percentage of conidia containing lipid droplets in wild-type 70-15, the Δ*Molfa1* mutant, and Δ*Molfa1-C* (*t*-test, * *p* < 0.1, ** *p* < 0.01). (**F**) The portion of appressoria containing lipid droplets in wild-type 70-15, the Δ*Molfa1* mutant, and Δ*Molfa1-C* (*t*-test, * *p* < 0.1, ** *p* < 0.01).

The conidia of *M. oryzae* contain large amounts of glycogen and lipid droplets. During conidial germination, glycogen and lipid droplets are transferred from the conidia to the appressoria, where they are absorbed and degraded to facilitate the host invasion process [39]. We hypothesized that MoLfa1 is involved in the utilization and degradation of glycogen and lipid droplets in *M. oryzae*. Therefore, we induced conidial germination on the surface of the hydrophobic membrane. KI/I_2_ and Bodipy were used to stain and observe the conidia, germ tubes, and appressoria induced after 0 h, 4 h, 8 h, 12 h, and 24 h of induction, respectively. As shown in Figure 6A,B, glycogen was abundant in the wild-type 70-15 and the Δ*Molfa1* mutant after 0 h of induction. After 4 h of induction, 68.28% of the wild-type 70-15 conidia contained glycogen, while 94.53% of the Δ*Molfa1* mutant conidia contained glycogen. After 8 h and 12 h of induction, the degradation rates of glycogen in wild-type 70-15 conidia were 48.19% and 93.62%, respectively, whereas the degradation rates of Δ*Molfa1* conidia were 32.73% and 83.25%, respectively. After 24 h of induction, 13.83% of the glycogen in the Δ*Molfa1* mutant appressoria was still not degraded. In contrast, glycogen was degraded in wild-type 70-15 appressoria (Figure 6C). The utilization and degradation of lipid droplets in the Δ*Molfa1* mutant showed the same trend as that of glycogen (Figure 6D–F). The above results indicate that MoLfa1 could inhibit the utilization and degradation of glycogen and lipid droplets in *M. oryzae*.

### 2.8. MoLfa1 Interacts with the RACK Protein MoMip11 and Is Involved in Mps1-MAPK Signaling Pathways in M. oryzae

**Figure 7:**
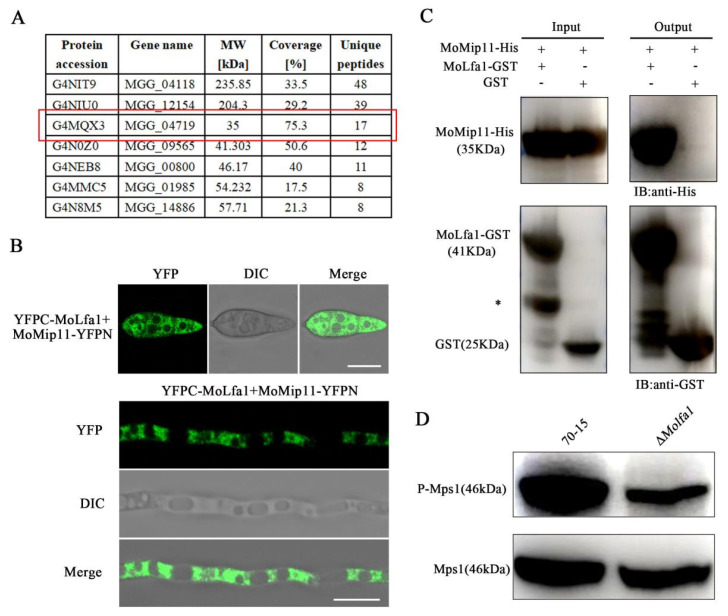
MoLfa1 interacts with the RACK protein MoMip11 in vivo and in vitro and is involved in Mps1-MAPK signaling pathways in *M. oryzae.* (**A**) Potential interacting proteins with MoLfa1 screened by mass spectrometry. The protein framed in red is the MoMip11. (**B**) BiFC assay to study the interaction between MoLfa1 and MoMip11 in vivo. A pair of YFPC-MoLfa1 and MoMip11-YFPN constructs was co-transformed into wild-type 70-15 by ATMT. YFP signals were detected using a Zeiss LSM880 confocal microscope, 63×oil objective. Bar, 10 μm. (**C**) Protein binding assay to detect the interaction between MoLfa1 and MoMip11 in vitro. GST protein or GST-tagged MoLfa1 protein was used to bind GST beads, and then His-tagged MoMip11 protein was incubated. His and GST antibodies were used for immunoblotting with the eluted solution. This * represents a non-specific protein. (**D**) The phosphorylation level of Mps1 in wild-type 70-15 and the Δ*Molfa1* mutant was determined.

To further investigate the function of MoLfa1, 7 proteins with high unique peptide values were selected as potential MoLfa1 interacting proteins by mass spectrometry (Figure 7A). To further explore the relationship between MoLfa1 and these proteins, we used BiFC to detect possible interactions between MoLfa1 and these proteins in vivo. We fused the N-terminal fragment of MoLfa1 with the pKD2-YFPN vector, the C-terminal fragment of MoMip11 with the pKD5-YFPC vector, and then transformed YFPN-MoLfa1 and MoMip11-YFPC into wild-type 70-15. As negative controls, we transformed the YFPC/YFPN-MoLfa1 and YFPN/MoMip11-YFPC constructs into wild-type 70-15. We detected YFP signals in transformants co-expressing YFPN-MoLfa1 and MoMip11-YFPC, whereas no such signals were detected in the negative controls, suggesting that full-length MoLfa1 interacts with MoMip11 in vivo (Figure 7B and Figure S3). In addition, we constructed *MoLFA1*-GST and *MoMIP11*-His vectors and expressed them in *Escherichia coli* BL21. Pull-down experiments showed that MoMip11-His interacts directly with MoLfa1-GST in vitro without interacting with empty GST (Figure 7C).

MoMip11 is a protein containing seven WD40 domains. It localizes in the ER [36] and interacts with Mst50 and Mck1 as a RACK protein in the Mps1-MAPK pathway [39]. To determine whether MoLfa1 plays a role in the Mps1-MAPK pathway, we measured the level of the Mps1 phosphorylation using anti-Mps1 antibodies. As shown in Figure 7D, the Mps1 phosphorylation level in the Δ*Molfa1* mutant is significantly reduced compared to the phosphorylation level in wild-type 70-15 strains. Taken together, we concluded that MoLfa1 promotes the phosphorylation level of Mps1 by interacting with MoMip11 and is involved in the Mps1-MAPK signaling pathway.

## 3. Discussion

Septins are thought to reorient and reorganize the cytoskeleton to determine cell shape and act as partitioning diffusion barriers to recruit and maintain specific proteins at discrete subcellular locations [1,40,41]. Studies have shown that the assembly of the septin ring is critical for the pathogenicity of *M. oryzae* [1,42]. However, the impact of septin ring development on the virulence of blast fungus remains uncertain. In this study, we found a protein, MoLfa1, which has a homologous protein only in pathogenic fungi such as *F. graminearum*, *F. oxysporum*, *B. graminis* f. sp. *tritici*, *Colletotrichum* spp. It has no homologous protein in yeast, humans, mice, drosophila, or nematodes. MoLfa1 can affect the absorption of LCFAs and associate with PIPs to affect the formation of the septin ring, thereby affecting the pathogenicity of *M. oryzae.* In addition, MoLfa1 is located in the ER and interacts with the ER-related protein MoMip11 to regulate the Mps1-MAPK pathway.

**Figure 8:**
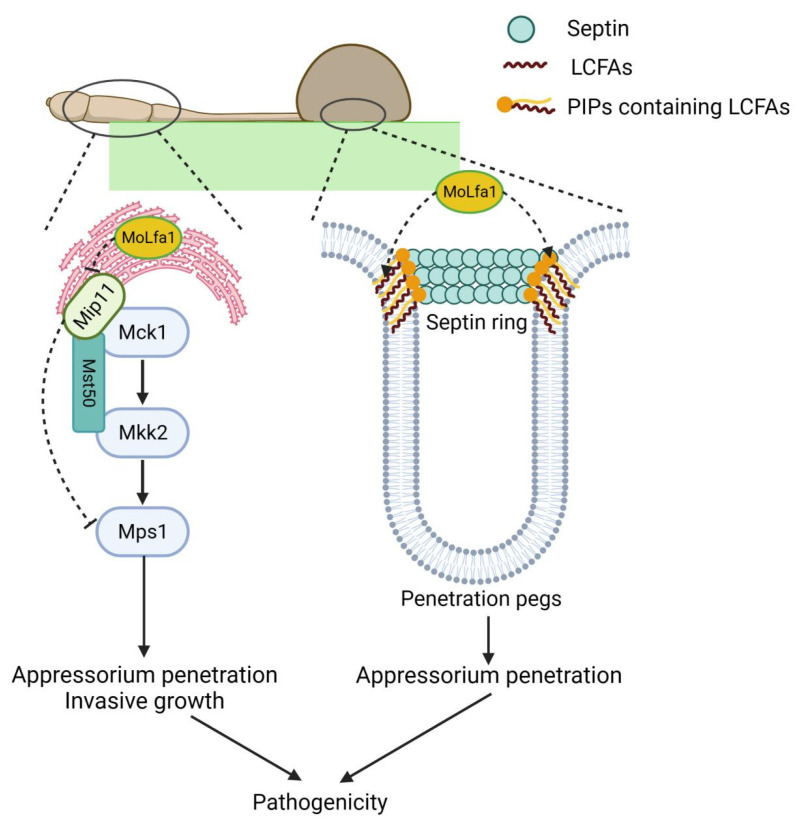
A proposed model of MoLfa1 in the regulation of *M. oryzae* virulence. MoLfa1 inhibits the uptake of LCFAs and associates with PIPs to participate in septin ring formation. In addition, MoLfa1 influences the phosphorylation level of Mps1 by interacting with MoMip11, thereby affecting the pathogenicity of *M. oryzae*.

LCFAs are the major components of PIPs, and PIPs belong to GPs. Recent studies have shown that LCFAs and PIPs are involved in forming the septin ring of *M. oryzae* appressoria [10]. In *M. oryzae*, the assembly of septin and the establishment of the F-actin network are necessary for *M. oryzae* to rupture the leaf cuticle of a plant [1]. The four guanosine triphosphatase membrane proteins assemble a ring F-actin network on the PIPs-rich membrane domain in the appressorium and polymerize into a dynamic heterooligomeric ring to infect rice [1,2,43]. LCFAs have unique biophysical properties, such as increasing the hydrophobicity of lipids, forming lipid blamers, and mediating the transition of lipids from the fluid to the gel phase. These properties contribute to the formation of highly curved membrane domains (penetration pegs) of *M. oryzae* [10]. MoSep3 is a core septin component of *M. orzyae*, essential for septin-ring assembly and host penetration. He et al. found that MoSep3 interacts with PIPs (such as PI3P, PI4P, PI5P, and PI(3,5)P2) of *M. oryzae* in vivo [10]. In this study, we found that MoLfa1 can also associate with PI3P, PI4P, and PI5P and inhibit the uptake of LCFAs to ensure normal septin ring formation. However, it is not clear why MoLfa1 does not associate with phosphatidylinositol diphosphate (PIP2) and phosphatidylinositol triphosphate (PIP3), which will be the direction of our next exploration. The absence of MoLfa1 disrupts intracellular LCFAs, inhibiting cell activity and septin ring formation. In this lipidomic analysis, most of the differential lipids between wild-type 70-15 and the Δ*Molfa1* mutant were GPs (about 61.69%). KEGG analysis showed that GPs metabolism was the most highly enriched pathway. In summary, MoLfa1 can regulate the LCFAs uptake and lipids metabolism of *M. oryzae* and associate with PIPs to regulate septin ring formation, thereby affecting the pathogenicity of *M. oryzae* (Figure 8).

MoMip11, an ER-localized RACK protein containing seven WD40 domains, interacts with MoLfa1, which is also ER-localized and identified in this study. Recent studies have shown that MoMip11 is involved in the Mps1-MAPK pathway by interacting with MoMck1 and MoMst50 [33]. In *M. oryzae*, the Δ*Momip11* mutant is sensitive to cell wall stress, similar to the homologous gene deletion mutant in *S. cerevisiae* and *U. maydis* [36]. In addition, the Mps1 phosphorylation level of Δ*Momip11* mutant was higher than that of wild-type strains [33]. Our study showed that the cell wall integrity of the Δ*Molfa1* mutant was unaffected. However, the phosphorylation level of Mps1 was significantly reduced in the Δ*Molfa1* mutant compared to the wild-type 70-15. This experimental result suggests that MoLfa1 interacts with MoMip11 to regulate the phosphorylation level of Mps1 in the Mps1-MAPK pathway, thereby affecting the pathogenicity of *M. oryzae* (Figure 8).

In conclusion, this study showed that MoLfa1 plays a critical role in mycelial growth, conidia development, appressorium formation, glycogen and lipid droplet utilization, lipid metabolism, and septin ring formation in *M. oryzae*. MoLfa1 regulates the formation of septin rings by inhibiting the uptake of LCFAs and binding with PIPs, thereby affecting the pathogenicity of *M. oryzae*. In addition, MoLfa1 interacts with the ER-related protein MoMip11 to regulate Mps1 phosphorylation levels to participate in the Mps1-MAPK pathway. MoLfa1 has homologous proteins in some pathogenic fungi, but no homologous protein in yeast, humans, or mice. Therefore, MoLfa1 is very suitable as a target for controlling pathogenic fungi such as *M. oryzae*. In the future, we should further clarify the role of MoLfa1 in septin ring formation and the utilization of LCFAs and develop new drugs for the prevention and treatment of rice blast based on the mechanism of MoLfa1.

## 4. Materials and Methods

### 4.1. Strains and Cultural Conditions

The strains used in this study included wild-type 70-15, *Escherichia ColiDH5α* (Trans T1, TransGen Biotech, Beijing China) and *Agrobacterium tumefaciens* (AGL1, Shanghai Weidi Biotechnology, Shanghai, China). Wild-type strain 70-15, the Δ*Molfa1* mutant, and the complemented strain Δ*Molfa1-C* were cultured on a CM medium) [44]. The growth test was carried out in a 7 cm medium at 25 °C (light for 16 h, dark culture for 8 h) for 8 days.

### 4.2. Gene Deletion and Complement Strategy

To obtain the Δ*Molfa1* mutant, we used the high-throughput gene knockout method invented by Professor Jianping Lu [37]. The knockout vector PKO3A containing the lethal gene *HSVtk* was isolated by restriction endonuclease *Xba*I and *Hind*III. Next, we used primers *MoLFA1* up (F)/*MoLFA1* up (R) and *MoLFA1* down (F)*/MoLFA1* down (R) to amplify the more than 1 kb upstream and downstream segments of *MoLFA1* in the *M. oryzae* genome (Table S1). Then, the amplified upstream and downstream fragments and a hygromycin-resistance gene (*HPH*) were fused with the above enzyme digestion vector PKO3A with multi-fragment fusion enzyme (Vazyme Biotech, Nanjing, China) and transferred into *Escherichia coli*. After a single colony was selected and verified to be correct, the plasmid was transferred into *Agrobacterium*. Finally, the wild-type 70-15 was transferred by the ATMT. The transformers were initially screened on the CM medium containing the hygromycin resistance (200 μg/mL), and then the knockout mutant strains were further verified by fluorescence and PCR methods (Figure S2).

To obtain the Δ*Molfa1-C* complement strain, we fused the full-length *MoLFA1* gene into a PKD5-GFP vector containing sulfonylureas resistance gene (*SUR*) and transformed it into the Δ*Molfa1* mutant by the ATMT method. Finally, the complement strains were identified by phenotypic recovery analysis and fluorescence observation. Similarly, the full-length Molfa1 gene was split into two segments, N1 (1–282 bp) and N2 (283–468 bp), then merged into the Δ*Molfa1* mutant to obtain the Δ*Molfa1-N1-C* and Δ*Molfa1-N2-C* strains.

### 4.3. Detection of Growth, Abnormal Conidia Rate, and Germination of M. oryzae

To detect the growth of *M. oryzae*, wild-type 70-15, the Δ*Molfa1* mutant, and complement strains Δ*Molfa1-C*, Δ*Molfa1-N1-C* and Δ*Molfa1-N2-C* were cultured on CM medium at 25 °C for 8 days (light 16 h, dark 8 h) [45]. The diameters of all strains were then measured and photographed separately. Conidia were collected with sterile water and stained with calcofluor white (CFW). Conidia were observed under a microscope, and the abnormal conidia were calculated. For conidia germination, conidia were collected with sterile water and diluted to 1 × 10^5^/mL. Then, 40 μL of suspended conidial droplets were placed on the surface of the hydrophobic membrane to induce conidial germination, which was incubated in a humidor at 28 °C. The morphology of the conidial droplets after 0 h, 4 h, 8 h, and 24 h of induction was observed under a microscope, and the germination rate was calculated [46].

### 4.4. Pathogenicity Analysis Experiment

For the barley pathogenicity experiment, the bacterial cakes of wild-type 70-15, the Δ*Molfa1* mutant, and complement strain Δ*Molfa1-C* grown for 8 days were placed on barley leaves (International Rice Research Institute, IRRI), developed for 7 days, and cultured at 25 °C for 4 days. Leaf emergence was then observed and photographed for preservation. Similarly, conidia were collected with sterile water and diluted to 1 × 10^5^/mL, and 20 μL suspended drops of conidia were applied to 7-day-old barley leaves. The infection of appressoria 48 h later and leaf disease 4 days later was observed [37]. Conidia were collected with sterile water and centrifuged for the rice spray experiment. The conidia concentration was 1 × 10^5^/mL with 0.25% gelatin (Sango Biotech, Shanghai, China). Two mL of conidial suspension was sprayed evenly on rice seedlings (CO-39; International Rice Research Institute, IRRI) and grown for 14 days. Disease was observed and photographed after 48 h of dark culture and then 4–5 days of alternating light and dark culture.

### 4.5. Degradation of Glycogen and Lipid Droplets

For the degradation of glycogen and lipid droplets, conidia were collected with sterile water and diluted to 1 × 10^5^/mL, and 40 μL of suspended conidia were placed on a hydrophobic membrane to induce the production of appressoria. KI/I_2_ and Bodipy were used to stain conidia, germ tubes, and appressoria after 0 h, 4 h, 8 h, 12 h, and 24 h of induction, respectively [39].

### 4.6. Subcellular Localization of MoLfa1

For the subcellular localization of *MoLFA1*, we constructed *MoLFA1*-mCherry expression vectors and transferred them into the Δ*Molfa1* mutant by ATMT transformation and then observed and photographed them under the microscope. To further determine the subcellular localization of MoLfa1-mCherry, we transferred the ER localization gene *MoSPF1*-GFP into the strain with *MoLFA1*-mCherry. Conidia were collected from the strains with sterile water to perform the above conidia germination experiment. Fluorescence microscopy was used to observe GFP and mCherry fluorescence of the conidia. The excitation wavelength of GFP is 488 nm and the emission wavelength is 520 nm. The excitation wavelength of mCherry is 558 nm and the emission wavelength is 583 nm.

### 4.7. Detection of M. oryzae Growth Rate in Different LCFAs Media

To detect the growth rate of *M. oryzae* in different LCFAs media, we replaced the 1% carbon source glucose in the substrate medium MM (the yeast extract, peptone 140, and casamino acid in CM medium were removed, and other components were the same as CM medium formula) with other LCFAs, including ferulic acid, tetradecanoic acid, lauric acid, palmitic acid, olive oil, oleic acid and tween 20 [22]. Growth rates of wild-type 70-15, the Δ*Molfa1* mutant, and complement strains in different carbon source media for 8 days were calculated using MM as the control medium.

### 4.8. Non-Targeted Lipidomics Assay

For non-targeted lipidomics analysis, wild-type 70-15 and the Δ*Molfa1* mutant were cultured in liquid CM medium at 25 °C for 2 days, harvested, and frozen in liquid nitrogen. We used a UPLC-MS/MS (QE Plus™, Shanghai Bioprofile Biotechnology Hangzhou, China) based liquid chromatography-mass spectrometry system to analyze the samples and MSDAIL software (Version 4.0.9) for lipid identification and data pre-processing [47].

### 4.9. Mass Spectrometry Experiment

For the mass spectrometry experiment, strains with MoLfa1-GFP were cultured in liquid CM medium at 25 °C for 2 days and total protein was extracted from the mycelium. Then 50 μL GFP glutathione agarose beads (Invitrogen, Waltham, MA, USA) and MoLfa1-GFP protein extract were incubated at 4 °C for 4 h. The eluents were analyzed by electrospray ionization mass spectrometry.

### 4.10. Septin Ring Observation and In Vitro Lipid Binding Experiment

For the septin ring observation experiment, we constructed the septin localization gene *MoSEP4*-GFP and transferred it into wild-type 70-15 and the Δ*Molfa1* mutant by the ATMT transformation method, and then observed and calculated their septin ring morphology under the microscope [10].

For the in vitro lipid binding experiment, we obtained purified MoLfa1-GST and GST protein solutions according to the prokaryotic expression protein induction and purification described in the pull-down experiment. PIP strips (Echelon Biosciences, Salt Lake City, UT, USA) containing different lipid components were immersed in 5% skim milk powder for 1 h, and then incubated in 5% skim milk powder containing the above purified protein (concentration 1 μg/mL) at 4 °C overnight. The PIP strips were washed three times with TBST for 10 min each time. The primary and secondary antibodies of GST were then incubated and developed according to the Western blot procedure [48].

### 4.11. In Vitro Protein Binding Experiments (Pull-Down)

To obtain the GST-MoLfa1 plasmid, we cloned the *MoLFA1* cDNA and ligated it into the pGEX4T-2 vector. First, we fused the full-length MoMip11 cDNA into the pET32a vector to obtain the MoMip11-His plasmid. We then transformed the plasmid DNA into *E. coli* BL21 (DE3) cells. Then, when the OD value of the bacterial solution was 0.4–0.6, IPTG was added (the final concentration was 0.4 mM) and cultured on a constant temperature shaking table at 18 °C at 150 rpm/min for 16 h. The cells were then harvested and treated with 150 mM NaCl, 10 mM Tris-HCl (pH 7.5), 0.5% Triton X-100, and 0.5 mM EDTA. Whole cell lysate was used for SDS-PAGE and then transferred to Coomassie blue solution for staining to confirm the expression of the resulting protein. After confirming normal expression, 50 μL of glutathione agarose beads (Invitrogen) were incubated with GST or MoLfa1-GST protein at 4 °C for 2 h and then with bacterial lysate containing MoMip11-His at 4 °C for 1 h. After incubation, the eluted proteins were detected by Western blot using anti-GST (HUABIO, Hangzhou, China) and anti-His antibodies (HUABIO, R1207-2).

### 4.12. Bimolecular Fluorescence Complementation Assay (BiFC) and Fluorescence Monitoring

The *MoLFA1* fragment was cloned into the pKD5-YFPC vector to generate the YFPC-MoLfa1 fusion construct. Similarly, the MoMip11 fragment was cloned into the pKD2-YFPN vector to generate the MoMip11-YFPN construct. A pair of YFPC-MoLfa1 and MoMip11-YFPN constructs was co-converted into wild-type 70-15 by ATMT. Strains resistant to both hygromycin and sulfonylureas were screened. The strain YFP signal was detected using a Zeiss LSM880 confocal microscope. The excitation wavelength of YFP is 488 nm and the emission wavelength is 520 nm.

### 4.13. Western Blot Analysis of Mps1 Phosphorylation

To detect the level of Mps1 phosphorylation, wild-type 70-15 and the Δ*Molfa1* mutant were cultured in liquid CM medium at 25 °C for 2 days, and total proteins were extracted from the mycelia. Then, we used an anti-phospho-p44/42 MAPK (T202/Y204) antibody (Cell Signaling Technology, Danvers, MA, USA) and an anti-p44/42 MAPK (Erk1/2) antibody (Cell Signaling Technology, Danvers, MA, USA) was used as a control.

## Data Availability

Data are contained within the article or Supplementary Materials.

## Supplementary Materials

The supporting information can be downloaded at: https://www.mdpi.com/article/10.3390/ijms25063434/s1.
