# Supplementary material for: The MoLfa1 Protein Regulates Fungal Development and Septin Ring Formation in Magnaporthe oryzae
Source: Int J Mol Sci. 2024 Mar 19;25(6):3434. doi: 10.3390/ijms25063434 (PMC10970683; doi:10.3390/ijms25063434)
Supplement: Supplementary file 1 [file ijms-25-03434-s001.zip › ijms-2859225-supplementary.pdf]

# Supplementary Material

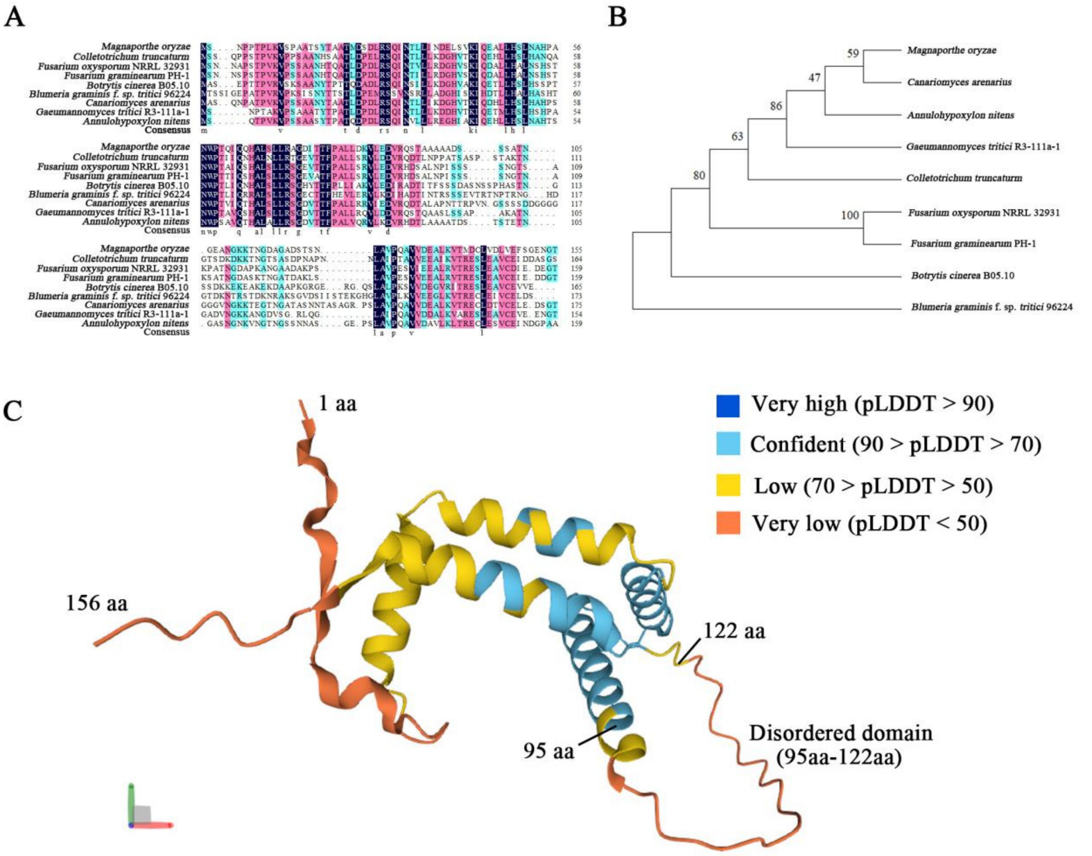


Fig. S1. Identification of MoLfa1 in *M. oryzae.* (A) CLUSTALW was used to align the amino acids of Lfa1 in *M. oryzae* (MoLfa1) with those of the homologous proteins in *C. truncatum* (XP_036575343.1), *F. oxysporum* NRRL 32931 (EWY86874.1), *F. graminearum* PH-1 (XP_011324077.1), *B. cinerea* B05.10 (XP_001553949.2), *B. graminis* f. sp. *tritici* 96224 (EPQ63018.1), *C. arenarius* (KAK4115313.1), *G. tritici* R3-111a-1 (XP_009217046.1), and *A. nitens* (KAI0901762.1). (B) MoLfa1 homology analysis in different species. (C) Molecular structure prediction of MoLfa1 protein. AlphaFold produces a per-residue confidence score (pLDDT) between 0 and 100. Some regions with low pLDDT may be unstructured in isolation. 95-122aa is a disordered region.

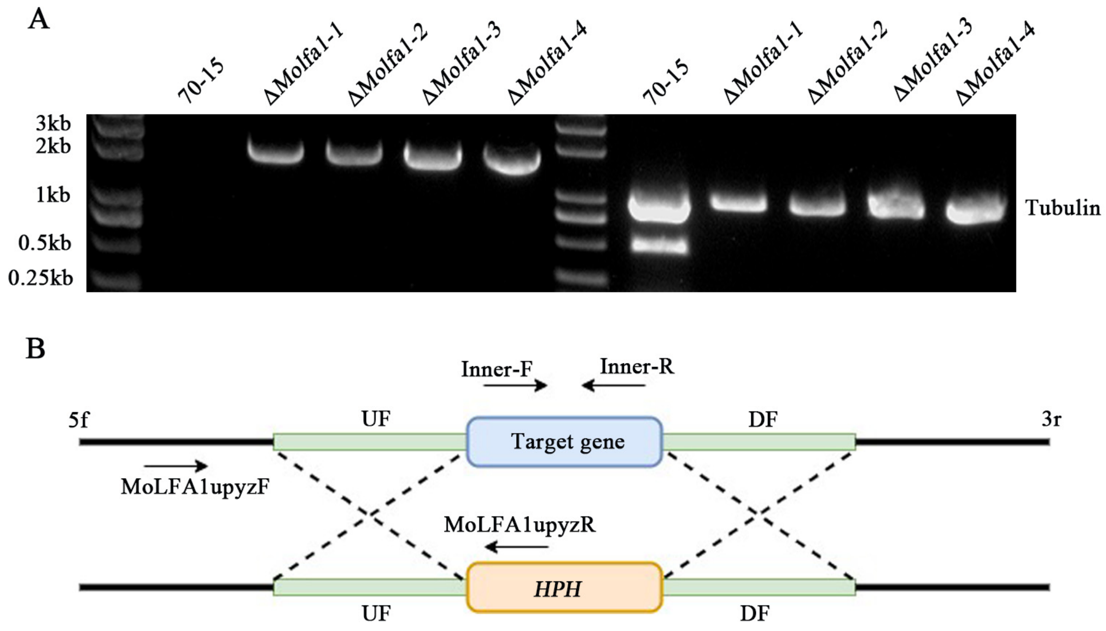


Fig. S2. The targeted gene in the transformant was screened by PCR using the tubulin gene as a positive control. A 0.5 kb short band was amplified from the wild-type strain and the ectopic transformant, indicating that the target gene was contained, whereas the 0.5 kb short band was not found in the null mutant. A unique recombinant DNA fragment labeled as a knockout event in the transformant was screened by PCR. A 1.5-2.5 kb long band was amplified from the null mutant, whereas the wild-type strain and the ectopic transformant did not have the long band. (B) The knockout model in *M. oryzae*.

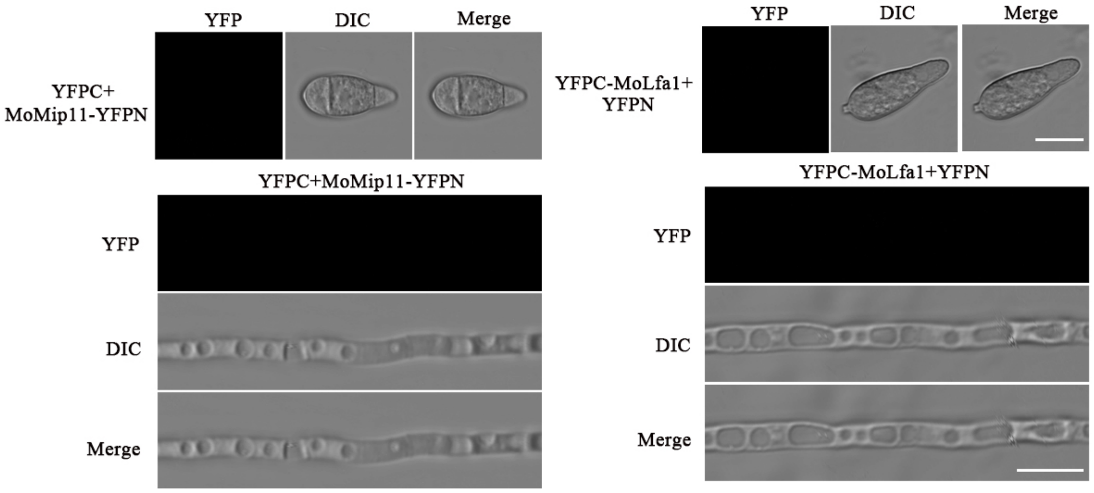


Fig. S3. A negative control of the interaction between MoLfa1 and MoMip11 in vivo.

Construct pairs of YFPC-MoLfa1 and YFPN, MoMip11-YFPN, and YFPC were co-transformed into wild-type 70-15 by ATMT to serve as negative controls. Bar, 10 μm.

| Name | Sequence (5’ - 3’) |
| --- | --- |
| Primers used for gene knockout | |
| HPH-F | TAGTGGAGGTCAACAATGAATG |
| HPH-R | CATCTACTCTATTCCTTTGCCC |
| MoLFA1up-F | AGGCTAACTGACACTCTAGAATGAGATGGAGGCGGTAT |

**Table S1 Primers in this study**

| | |
|---|---|
| MoLFA1up-R | TGTTGACCTCCACTAACAGAAAGAGGAAGGCAGT |
| MoLFA1dn-F | GGAATAGAGTAGATGACCTCATTCGAGGCCCTATGC |
| MoLFA1dn-R | CGACGGCCAGTGCCAAGCTTGATGAAGAATGCTGGCCC |
| MoLFA1inner-F | TCCGGCGGCGACAAGTTAT |
| MoLFA1inner-R | CGACTAAATCGACTAAGCAG |
| MoLFA1upyz-F | CCGACAAAGCTTTCCTGCAC |
| MoLFA1upyz-R | GTCGGAGACGCTGTCGAACTT |
| Tubulin-F | CCATCCCGAGCTTGTTGATA |
| Tubulin-R | GTAGTTCAGGTCACCGTATGAG |
| Primers used for complementation | |
| pKD5-MoLFA1-GFP-F | ATCACAATGGCCGGATCCATGTCAAACCCACCGACACCG |
| pKD5-MoLFA1-GFP-R | CTTGCTCACCATCCCGGGAGTAGTGCCATTCTCCCCCGA |
| pKD5-MoLFA1-N1-GFP-F | ATCACAATGGCCGGATCCATGTCAAACCCACCGACACC |
| pKD5-MoLFA1-N1-GFP-R | CTTGCTCACCATCCCGGGCGCAGTGCTCTGCCTGACGT |
| pKD5-MoLFA1-N2-GFP-F | ATCACAATGGCCGGATCCGCGGCGGCGGCGGATTCATC |
| pKD5-MoLFA1-N2-GFP-R | CTTGCTCACCATCCCGGGAGTAGTGCCATTCTCCCCCGA |
| Primers used in fluorescent observation | |
| MoLfa1-mCherry-F | ATCACAATGGCCGGATCCATGTCAAACCCACCGACACCG |
| MoLfa1-mCherry-R | CTTGCTCACCATCCCGGGAGTAGTGCCATTCTCCCCCGA |
| Primer used for Pull-down | |
| MoLfa1-GST-F | CTGGTTCCGCGTGGATCCATGTCAAACCCACCGACACCG |
| MoLfa1-GST-R | CGGGAATTCCGGGGATCCAGTAGTGCCATTCTCCCCCGA |
| MoMip11-His-F | CAAGGTCGACAAGCTTATGGCTGAGCAGCTCATT |
| MoMip11-His-R | GTGCGGCCGCAAGCTTTGCCCTAGACATGACGCC |
| Primer used for BiFC | |
| YFPC-MoLfa1-F | GAGCTGTACAAGTCTAGAATGTCAAACCCACCGACACCG |
| YFPC-MoLfa1-R | CGCTTACTGCAGGTCGACAGTAGTGCCATTCTCCCCCGA |
| MoMip11-YFPN-F | GTCAAAATGGTCGGATCCATGGCTGAGCAGCTCATT |
| MoMip11-YFPN-R | GGCGATGGAGCGCCCGGGTGCCCTAGACATGACGCC |
